# Metabolic tumor parameters complement clinicopathological factors in prognosticating advanced stage Hodgkin Lymphoma

**DOI:** 10.22038/AOJNMB.2023.69260.1482

**Published:** 2023

**Authors:** Ashish Mohite, Venkatesh Rangarajan, Jayant Goda, Swati Chugh, Archi Agrawal, Manju Sengar

**Affiliations:** 1 Department of Nuclear medicine and Molecular Imaging, Tata Memorial Centre, Mumbai Maharashtra, India; 2 Department of Radiation Oncology, Tata Memorial Centre, Mumbai Maharashtra, India; 3 Department of Haematooncology, Tata Memorial Centre, Mumbai Maharashtra, India; 4 Homi Bhabha National Institute, Anushaktinagar, Mumbai, Maharashtra, India

**Keywords:** Advanced Hodgkin’s lymphoma, 18F-FDG PET/ CT, Metabolic tumor parameters, Clinicopathological parameters, Event free survival

## Abstract

**Objective(s):**

Advanced Hodgkin Lymphoma has a higher probability of relapse and recurrence. Classical clinicopathological parameters including the International Prognostic Score (IPS) have not been reliable in predicting prognosis or tailoring treatment. Since FDG PET/CT is the standard of care in staging Hodgkin Lymphoma, this study attempted to evaluate the clinical utility of baseline metabolic tumor parameters in a cohort of advanced Hodgkin lymphoma (stage III and IV).

**Methods:**

Histology-proven advanced Hodgkin Patients presenting to our institute between 2012-2016 and treated with chemo-radiotherapy (ABVD / AEVD) were followed up till 2019. Quantitative PET/CT and clinicopathological parameters were used to estimate the Event Free Survival (EFS) in 100 patients. Kaplan-Meier method with log-rank test was used to compare the survival times of prognostic factors.

**Results:**

At a median follow-up of 48.83 months (IQR:33.31-63.05 months), the five-year-EFS was 81%. Of the 100 patients, 16 had relapsed (16%) and none died at the last follow-up. On Univariate analysis, among non-PET parameters bulky disease (P=0.03) and B-symptoms (P=0.04) were significant while among PET/CT parameters SUV_max_ (p=0.001), SUV_mean_ (P=0.002), WBMTV2.5 (P<0.001), WBMTV41% (P<0.001), WBTLG2.5 (P<0.001) and WBTLG41% (P <0.001) predicted poorer EFS. 5-year EFS for patients with low WBMTV2.5 [<1038.3 cm3] was 89% and 35% for patients with high WBMTV2.5 [≥1038.3 cm3] (p <0.001). In a multivariate model, only WBMTV2.5 (P=0.03) independently predicted poorer EFS.

**Conclusion:**

PET-based metabolic parameter (WBMTV2.5) was able to prognosticate and complement the classical clinical prognostic factors in advanced Hodgkin Lymphoma. This parameter could have a surrogate value for prognosticating advanced Hodgkin lymphoma. Better prognostication at baseline translates to tailored or risk-modified treatment and hence higher survival.

## Introduction

 Efforts of various international cooperative groups over the last three decades have made Hodgkin Lymphoma a potentially curable malignancy. Approximately 90% of the patients with early-stage disease are cured when treated optimally; combined modality treatment of chemotherapy and radiotherapy can cure 65-85% of patients with the advanced-stage disease (1). As per data from the National Cancer Institute (NIS) mean 5-year relative survival (2013-2017) was 87.4% (all stages

combined); being lowest for stage IV, 76.3% (2). 

 International Prognostic Score (IPS) has been widely used for predicting outcomes in advanced Hodgkin lymphoma patients, but it fails to identify a group of patients at high risk of treatment failure given the underrepresentation of patients with high IPS (≥5) which is only 7% (3).

 The advent of PET / CT as a staging and response evaluation modality in Hodgkin lymphoma has resulted in a paradigm shift in treatment strategies and prognosticating of the disease and has proven to be a superior modality to the conventional IPS scoring system (4-6). FDG PET / CT has withstood the test of time as a staging and response evaluation modality of choice in high-grade lymphomas (7, 8). PET / CT response-based chemo-radiotherapy has improved outcomes and has made tailored treatment a reality (9-13). 

 However, additional information can be derived from staging PET scans in terms of metabolic tumor burden. It is suggested that tumor burden in Hodgkin lymphoma correlates with prognosis (14, 15). In contrast to staging CT scan, PET / CT apart from accurately estimating the tumor burden also provides additional information on the aggressiveness of the tumor cells and hosts reactivity against the tumor (16). Although conventional metabolic parameters like SUV_max_ can be used to prognosticate Hodgkin lymphoma it varies with histological subtypes (17) and is limited by the single voxel value. Volumetric metabolic parameters like metabolic Tumor Volume (MTV) and Total Lesion Glycolysis (TLG) have an inherent advantage of not only representing the whole-body tumor burden but also being derived conveniently from the PET / CT scan.

 Although multiple studies have shown the prognostic value of both conventional (SUV_max_) and volumetric metabolic parameters (MTV and TLG) in prognosticating early-stage disease (18-24), controversy still exists regarding the prognostic significance of these parameters in advanced-stage Hodgkin lymphoma. Literature studies that investigated the role of these PET parameters, showed heterogeneous results (22, 25) which could be attributed to the lack of uniform treatment protocols or varied volume calculation techniques. In the context of these observations, we sought to explore the role of baseline ^18^F FDG PET / CT parameters as a measure of total tumor burden to better prognosticate patients with advanced Hodgkin lymphoma, uniformly treated with ABVD chemotherapy (Doxorubicin, Bleomycin, Vinblastine, Dacarbazine) and radiotherapy.

## Methods

***Patient cohort***

**Figure 1:**
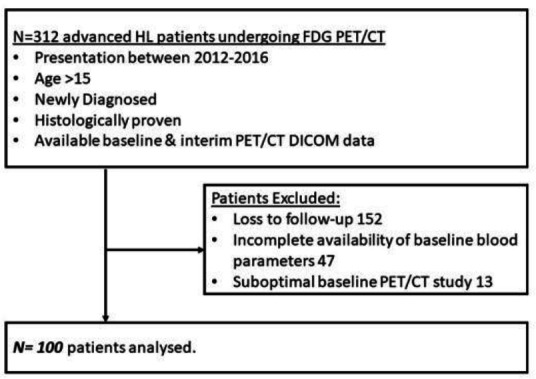
Flow chart of patient selection

 We conducted a retrospective chart review of one-hundred patients aged 15 years and above, diagnosed with stage III or IV Hodgkin between 2012 and 2016 and treated at our institute. The study was initiated after the approval from the institutional review board. All patients had baseline and interim PET/CT for staging and response evaluation post 2-4 cycles of ABVD or AEVD chemotherapy and radiotherapy based on institutional protocol. Those patients in whom baseline PET / CT DICOM data and the essential clinicopathological parameters were available in hospital records were selected (Figure 1). Clinicopathological parameters including age, sex, B group of symptoms and IPS were evaluated. Written, informed consent was waived, as this was a retrospective study and data was collected from hospital medical records.

***Imaging protocol and Image analysis***

 After a fasting period of 6 hours, patients were injected with 5 MBq/kg of ^18^F-FDG and scanned after 45 to 60 minutes with Philips Gemini-TF time of flight (TOF) 16/64 slice PET/CT scanners. Initial low dose whole-body CT scan was acquired followed by a PET scan. CT was used for PET attenuation and anatomical localization. Bed position was acquired for 60-90 seconds each. PET was acquired in 3D mode.

 Nodal lesions showing increased uptake over the background were considered as involved in Lymphoma. Lymph nodes ≥7cm were considered bulky. Extranodal lymphoma sites such as lung, liver, and bone marrow were delineated if they showed focal hyper- metabolism. The homogenous pattern of increased uptake within bone marrow was not included. Spleen involve-

ment was considered if it showed focal or diffuse hypermetabolism, clearly above the liver background.

**Figure 2:**
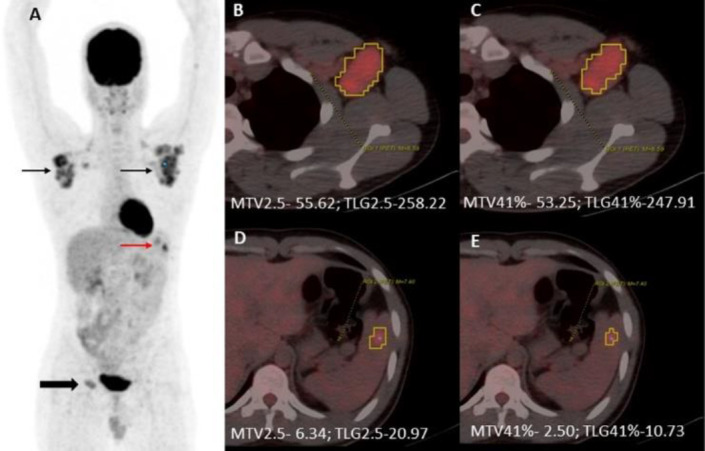
MTV and TLG calculation in a representative patient with advanced stage Hodgkin Lymphoma. (**A**) MIP image depicts FDG avid bilateral axillary (**black arrows**), inguinal (**block black arrow**) adenopathy, along with splenic lesion (**red arrow**). Fused PET/CT images depict MTV and TLG calculation of the left axillary node with a fixed cut-off of 2.5 (**B**) and percentage cut-off of 41% (**C**). Again, fused PET/CT images are depicting MTV and TLG determination of focal splenic lesion with a fixed cut-off of 2.5 (**D**) and a percentage cut-off of 41% (**E**). Abbreviations: MIP, Maximum Intensity Projection; FDG, Fluorodeoxyglucose; PET/CT Positron Emission Tomography/ Computed Tomography; MTV, Metabolic Tumor Volume; TLG, Total Lesion Glycolysis

 The VOI was identified by drawing spheres or cubes around each focus of increased uptake on the disease site individually; care was taken to incorporate the lesion in all planes. The software then automatically measured the SUV_max_, SUV_mean_, MTV, and TLG (Figure 2). 

 MTV and TLG were calculated using the software PET VCAR (Volume Computed Assisted Reading) from GE Healthcare. MTV was measured using the fixed and percentage threshold method, as recommended by EANM (26), and published in various lymphoma subtypes (12.23, 27-30) i.e., SUV_max_ threshold of 2.5 (MTV2.5) and 41% of the SUV_max_ (MTV41%) within the involved site. TLG was calculated as SUV_mean_ times the corresponding MTV - TLG2.5 and TLG41%. Whole-body metabolic tumor volume (WBMTV) and whole-body total lesion glycolysis (WBTLG) were nothing but the sum of the individual MTV and TLG.

***Treatment details and follow-up evaluation***

 Patients were treated with ABVD or AEVD chemotherapy regimens. Bleomycin was replaced by etoposide in patients whose compromised pulmonary functions. Indications for RT included bulky disease at presentation, extra-nodal disease, and residual disease in interim PET. RT was delivered by involved ﬁeld radiotherapy (IFRT) or involved site radiotherapy 

(ISRT). The planned dose of RT was 25.2 Gy/14 fractions for complete metabolic responders (Deauvelle’s:1-2) on interim PET while patients who had PR (Deauvelle’s:3-4) on iPET received 34.2 Gy/19 fractions as per institutional protocol. 

 After completion of treatment, patients were followed-up at regular intervals. During follow-up, patients with suspicion of relapse underwent restaging with PET/ CT. All relapses were confirmed with a biopsy. 

***Statistical Analysis***

 Demographic and tumor parameters were analyzed using descriptive statistics. EFS was calculated from date of diagnosis to detection of local or distant failure/development of secondary malignancy, date of last follow up or death whichever occurred first. Relapse was defined as recurrence after achieving complete remission. Continuous variables were categorized based on the optimal cut-off derived from the ROC curve using Youden’s index, except age which was categorized based on the median. Univariate analysis was done to 

assess the association of various prognostic factors such as age, sex, stage, presence or absence of ‘B’ symptoms and bulky disease, IPS, SUV_max_, SUV_mean_, WBMTV2.5, WBMTV41%, WBTLG2.5 and WBTLG41% with EFS. Kaplan-Meier method with log-rank test was used to compare the survival times of prognostic factors. Multivariate cox regression was performed to select disease characteristics that were statistically significant on univariate analysis. Statistical significance was assumed at P<0.05. Analysis was carried out using Statistical package for social sciences (SPSS) version 21 and R Studio Version 1.1.15.

## Results

***Patient Characteristics***

 A total of 312 newly diagnosed advanced Hodgkin lymphoma patients who underwent baseline PET/CT between 2012- 2016 were screened. 212 patients were excluded, as they were lost to follow-up (152), incomplete blood parameters (47) and due to suboptimal PET study (13); the remaining 100 patients formed the study cohort. The median follow-up was 48.83 months, (IQR: 33.31-63.05). Patient demographics and tumor parameters are summarised in Table 1. At the last follow-up, 16 patients had relapsed. Event-free survival at 5 years was 81%.

**Table 1:** Patient demographic and tumor profile (N=100)

**Characteristics**	**Frequency (%)**
**Age(years) median**	**30 years (IQR: 21-41.65)**
**Gender**	
Male	69 (69%)
Female	31 (31%)
**Histological variants**	
Lymphocyte Rich	3 (3%)
CHL- Mixed Cellularity	26 (26%)
CHL- Nodular Sclerosis	27 (27%)
Not classified	36 (36%)
Nodular Lymphocyte Predominant	8 (8%)
**Stage**	
III	52 (52%)
IV	48 (48%)
**Bulky Disease**	
Yes	39 (39%)
No	61 (61%)
**‘B’ Symptoms**	
Yes	62 (62%)
No	38 (38%)
**International Prognostic Score (IPS)**	
Low risk (0-2)	44 (44%)
Intermediate risk (3-4)	49 (49%)
High risk (5-7)	7 (7%)

Abbreviations: IQR, Inter-Quartile Range

***Assessment of baseline clinical parameters for event-free survival***

**Figure 3:**
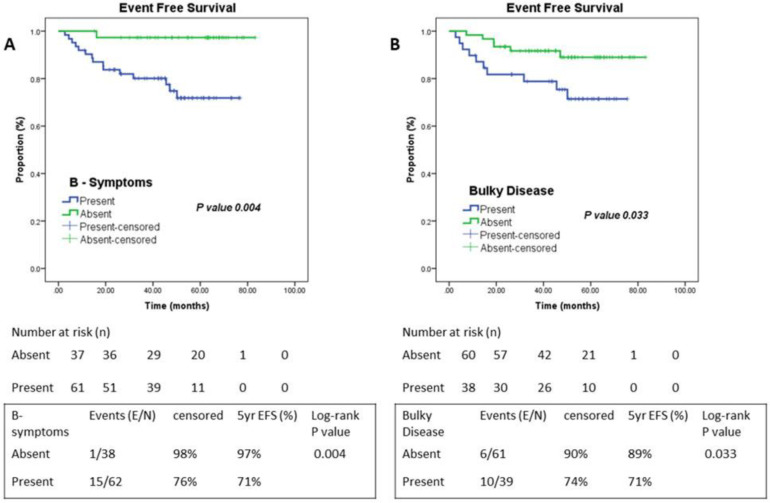
Kaplan- Meier survival curves of B-symptoms and Bulky disease. (**A**) B-Group of Symptoms and (**B**) Bulky Disease

Abbreviations: E/N – Events/ Total; 5yr EFS- 5-year Event Free Survival

 B-symptoms were present in 62/100 patients (62%). The 5-year EFS in patients who had B symptoms was 71% vis-a-vis 97% in patients without B symptoms (P=0.004). The bulky disease was observed in 39 patients. Patients who had bulky disease had worse 5-year EFS (71%), compared to patients with non-bulky disease (89%) (P=0.033). A significant number of events were associated with B-symptoms (E/N; 15/62) and Bulky disease (E/N; 10/39) (Figure 3). 

***Assessment of baseline metabolic PET parameters and correlation***

**Figure 4:**
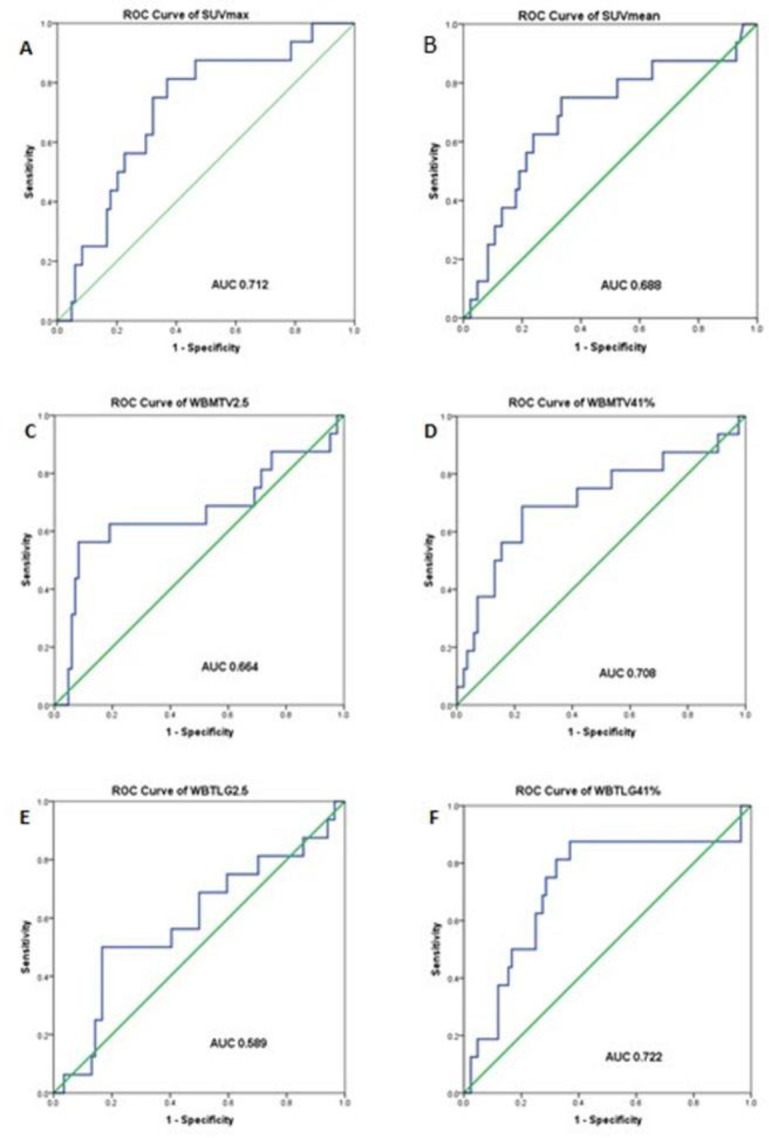
Receiver Operating Characteristic Curve (ROC) of multiple PET parameters. (**A**) SUV_max_ with AUC 0.712, (**B**) SUV_mean_ with AUC 0.688, (**C**) WBMTV2.5 with AUC 0.664, (**D**) WBMTV41% with AUC 0.708, (**E**)WBTLG2.5 with AUC 0.589 and (**F**) WBTLG41% with AUC 0.722.

Abbreviations: AUC, Area Under Curve; SUV, Standardised Uptake Value; MTV, Metabolic Tumor Volume; TLG Total Lesion Glycolysis

 The best cut-off for continuous PET/CT variables was derived from the ROC curve using Youden’s Index (Figure 4 and Table 2). 

**Figure 5:**
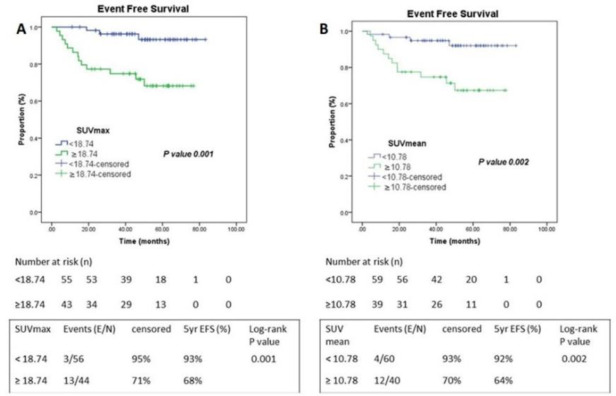
Kaplan Meier Curve of SUV_max_ and SUV_mean_. (**A**) SUV_max_ and (**B**) SUV_mean_. Abbreviations: E/N – Events/ Total; 5yr EFS: Five-year Event Free Survival

 Analyzing the conventional PET/CT parameters - SUV_max_ and SUV_mean_, they had AUC of 0.712 and 0.688 respectively while their optimal cut-off from the Youden’s index were 18.74 and 10.78. Patients with higher SUV_max_ (≥18.74) had 5-year EFS of 68% vis-a-vis 93% in those with lower SUV_max_ (P=0.001). Individuals with SUV_mean_ <10.78 had better 5-year EFS of 92% in comparison to those with higher SUV_mean_ ≥10.78, 64% (P=0.002) (Figure 5). 

**Table 2:** Threshold values of various Metabolic PET parameters

**Metabolic Parameters**	Threshold values*	**Sensitivity %**	**Specificity %**
SUV_max_ (g/ml)	18.74	81	64
SUV_mean_ (g/ml)	10.78	75	67
WBMTV2.5 (cm3)	1038.37	56	92
WBMTV41% (cm3)	289.39	69	78
WBTLG2.5 (g/ml×cm3)	4255.89	50	84
WBTLG41% (g/ml×cm3)	1271.77	87	64

Abbreviations: SUV, Standardized uptake value; WBMTV, Whole Body Metabolic Tumor Volume; WBTLG, Whole Body Total Lesion Glycolysis.

*Threshold values were derived from Receiver Operator Characteristics (ROC) Curve using Youden’s Index

**Figure 6:**
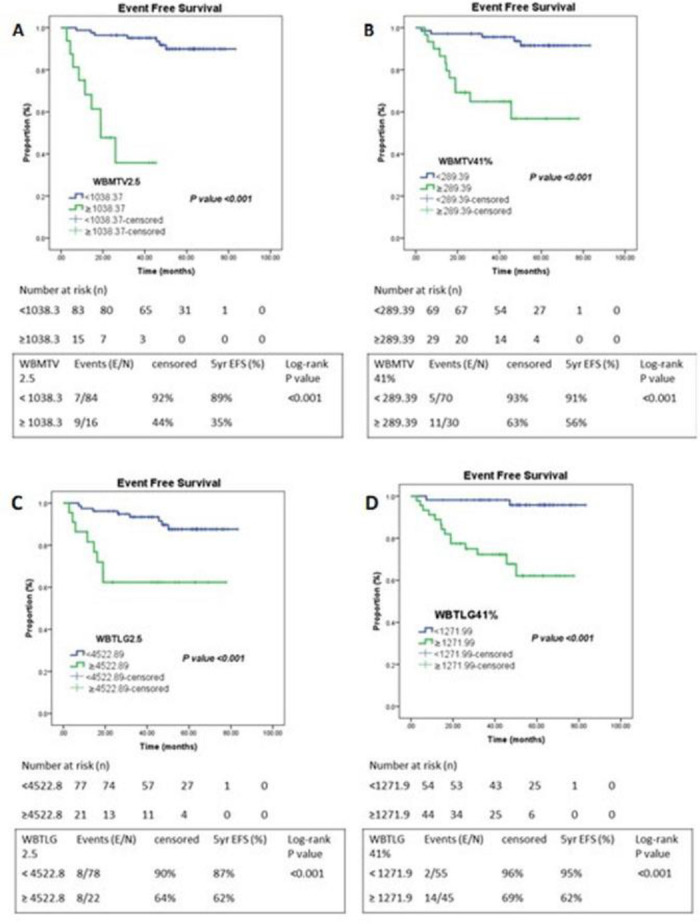
Kaplan Meier Curve of metabolic volume PET parameters. **A**) WBMTV2.5, **B**) WBMTV41%, **C**) WBTLG2.5, **D**) WBTLG41%.

Abbreviations: E/N, Events/Total; 5-yr EFS, 5-year Event Free Survival

We also studied the volume-based parameters, namely WBMTV2.5 and WBMTV41%, WBTLG2.5 and WBTLG41% respectively. AUC for WBMTV2.5 was 0.664 and optimal cut-off by Youden’s index from the ROC was 1038.37; Patients with higher WBMTV2.5 than the threshold of 1038.37 had a worse outcome in terms of 5-year EFS of only 35% as compared to patients whose WBMTV2.5 was less than 1038.37; 5-year EFS: 89% (P<0.001) (Figure 6A). The AUC of WBMTV41% was 0.708 and the threshold cut-off value of 289.39. Patients with higher than the threshold (≥289.39; n=30) had significantly poorer outcomes (5-year EFS: 56%) as opposed to patients who had lower than cut-off (<289.39; n=70) with a 5-year EFS of 91% (P <0.001) (Figure 6B).

WBTLG is another volumetric parameter that has been studied extensively in prognosticating lymphomas. We observed that the AUC for WBTLG2.5 and WBTLG41% to be 0.58 and 0.72 respectively; while their optimal cut-off values were 4255.89 and 1271.99 respectively as derived from the ROC. Dichotomizing these volumetric parameters based on their cut-off value, it was evident that patients with lower TLG fared much better as compared to those with higher values (WBTLG2.5 P <0.001; WBTLG41% P <0.001). Patients with higher WBTLG2.5 had a worse 5-year EFS of 62% vis-a-vis patients with lower WBTLG2.5 (5-year EFS: 87%). Those with a higher WBTLG41% again had inferior outcomes (5-year EFS: 62%) while those patients who had lower value had better clinical outcomes (5-year EFS: 95%) (Figure 6C and 6D). 

***Univariate analysis of clinical and metabolic parameters for EFS***

 Univariate cox regression was performed for all potential risk factors impacting disease relapse including patient-related and tumor-based parameters. Among the patient and tumor-related parameters, the presence of B-symptoms was statistically significant for poorer clinical outcomes (P=0.04; HR 10.811)

while age, gender, stage of lymphoma, and Hasenclever index did not have any impact on EFS on univariate cox regression analysis. Among the tumor related parameters, the bulky disease was associated with inferior clinical outcomes (P=0.033; HR 2.866). Assessment of conventional metabolic parameters, SUV_max_ (P=0.001; HR 6.172) and mean (P=0.002; HR 4.923) reliably predicted for 5-year EFS. Univariate analysis of all the volume-based metabolic parameters (WBMTV2.5, WBMTV41%, WBTLG2.5, WBTLG41%) also predicted the 5-year clinical endpoint (Table 3).

**Table 3:** Univariate Cox Regression analysis of the patient and tumor-based parameters for event free survival

**Clinical and metabolic Parameters**	**Hazard Ratio**	** 95% CI**	**P value**
Gender	0.992	0.344-2.859	0.988
Age	0.445	0.154-1.286	0.125
Stage	1.507	0.561-4.047	0.413
B- Symptoms	10.811	1.427-81.934	0.004
IPS≥2	1.175	0.335-4.130	0.800
IPS≥3	1.104	0.410-2.969	0.845
Bulky Disease	2.866	1.041-7.892	0.033
SUV_max _(≥18.74)	6.172	1.758-21.667	0.001
SUV_mean_ (≥ 10.78)	4.923	1.587-15.271	0.002
WBMTV 2.5 (≥1038.37)	20.041	5.993-66.992	<0.001
WBMTV 41% (≥289.39)	7.727	2.622-22.774	<0.001
WBTLG 2.5 (≥4255.89)	4.976	1.853-13.362	<0.001
WBTLG 41% (≥1271.99)	11.623	2.618-51.605	<0.001

Abbreviations: CI, Confidence Interval; IPS, International Prognostic Score; SUV, Standardized uptake value; WBMTV, Whole Body Metabolic Tumor Volume; WBTLG, Whole Body Total Lesion Glycolysis

***Multivariate analysis of clinical and PET-based metabolic parameters for EFS***

 WBMTV2.5 was the only parameter which retained statistical significance and independe-

ntly predicted for 5-year EFS on the multivariate cox regression model [P=0.030, HR 7.961 (95% CI: 1.223-51.824)], the hazard ratio and 95 % CI of all the prognostic factors are summarised in Table 4.

**Table 4:** Multivariate Cox Regression Analysis of the prognostic factors for event free survival

**Factors**	**Hazards Ratio**	**(95% CI)**	**P-value**
Bulky Disease	0.396	0.111-1.411	0.153
B -symptoms	0.129	0.015-1.669	0.058
SUV_max_ (≥18.74)	3.187	0.447-22.719	0.247
SUV_mean_ (≥ 10.78)	1.897	0.321-11.205	0.480
WBMTV 2.5 (≥1038.37)	7.961	1.223-51.824	0.030
WBMTV 41% (≥289.39)	2.372	0.362-15.554	0.368
WBTLG 2.5 (≥4255.89)	0.842	0.257-2.761	0.777
WBTLG 41% (≥1271.99)	2.264	0.365-14.024	0.380

Abbreviations: CI, Confidence Interval; IPS, International Prognostic Score; SUV, Standardized uptake value; WBMTV, Whole Body Metabolic Tumor Volume; WBTLG, Whole Body Total Lesion Glycolysis

## Discussion

 This study shows the prognostic value of baseline WBMTV over and above the traditional and widely used IPS in a homogenously treated cohort of advanced Hodgkin lymphoma patients. The evidence for WBMTV as a prognostic marker has been proven from the subgroup analysis of the GHSG HD10 study (27) and various other studies in early-stage Hodgkin lymphoma (18,19) however, in the advanced stage, its role has not been very well documented (25). A cohort of 267 patients with early-stage unfavourable Hodgkin, MTV, and TLG as delineated by manual segmentation and sub-thresholding with SUV_max_ ≥2.5 could provide a reliable measure of the tumor burden that could be used as a potential aid to risk stratify patients (18). 

 According to the Survival Epidemiology and End Result (SEER) data, survival in early stages is around 90-95% but in advanced-stage disease, it declines to 84% in stage III and 75% in stage IV with the standard chemotherapy regimen and consolidation radiotherapy (2). 

 Approximately 15% of patients relapse within 5-years in the advanced stage (31). Therefore, prognostic factors are necessary to identify patients at low or high risk to avoid relapse and accordingly tailor therapy. Clinical prognostic factors have been developed to risk-stratify patients in both early and advanced stage Hodgkin (32). The Hasenclever-IPS is the most widely used scoring system for predicting outcomes in advanced Hodgkin lymphoma patients, but this scoring system although pragmatic fails to identify a sub-group of patients who may be at high risk for treatment failure (3), it is thus important to identify factors which would risk stratifying these patients. ^18^F-FDG PET/CT has been considered the most powerful investigation to calculate tumor burden, which in turn is the most important prognosticator of Hodgkin (15). 

 However, its utility had not been explored to adapt the treatment in advanced stage Hodgkin lymphoma. Therefore, we attempted to look at its added clinical utility derived from the metabolically active tumor parameters from 

baseline PET CT in advance Hodgkin lymphoma treated with combined chemotherapy (ABVD) and consolidation RT.

 The observations from our study showed the clinical utility of MTV in risk stratifying these patients in terms of 5-year EFS (patients with high MTV volume ≥1038cm3 had lower 5 years EFS). Among the PET/CT parameters, Whole Body Metabolic Tumor Volume with a fixed SUV_max_ cut-off of 2.5 (WBMTV2.5) proved superior to SUV_max_, SUV_mean_, WBMTV41% and WBTLG. A threshold cut-off volume of 1038 cm3 as obtained from the ROC curve using Youden’s index was able to risk stratify and prognosticate the disease. Among the patients who relapsed (16/100), nine had WBMTV2.5 ≥1038.37cm3 (E/N -9/16). Patients having WBMTV2.5 ≥ 1038.37cm3 had a significantly lower 5-year EFS (35%) compared to those with WBMTV2.5 <1038.37cm3 (89%) (P <0.001).

 Disease burden determined by PET CT recapitulates not only tumor spread but also reflects tumor aggressiveness and the host’s reactivity against the tumor (33). The initial evidence of tumor burden being a strong prognostic factor was demonstrated by Specht et al. (34) and confirmed by Gobbi et al., 13 years later (35). However, both these studies were based on morphological CT based images. It is a well-known fact that the predominant neoplastic component in Hodgkin resides in the surrounding microenvironment of inflammatory and accessory cells with less than 1-2% of Reed Sternberg cells. Therefore, PET better quantitates the tumor burden by estimating the functional tumor volume, a better reflection of the infiltrating microenvironment. Our results, clearly demarcate the superiority of Metabolic FDG PET/CT parameters (SUV_max_, SUV_mean_, WBMTV and WBTLG) over conventional clinicopathological and radiological features.

 Contemporary trials investigating the various combinations of chemotherapy regimens with or without radiotherapy in advance Hodgkin lymphoma have used PET response to adapt the therapy (11, 36, 37). The RATHL trial (CRUK/07/033) looked at the metabolic parameters (MTV and TLG) as a prognostic marker in a cohort of 100 patients and suggested that MTV and TLG using a cut-off of >2.5 significantly predicted survival, however, a cut-off using 41% did not have any prognostic significance; however, when these metabolic parameters were reanalyzed in a larger cohort of 484 patients, only TLG2.5 predicted for the survival outcomes (38). The results of our study corroborated well with the initial observations from the RATHL trial. The AHL2011 LYSA study also analyzed the baseline metabolic parameters in a cohort of 392 patients with advanced Hodgkin lymphoma (IIB-IV). The results of this study showed that baseline WBMTV41% in combination with interim PET could identify a subset of Hodgkin lymphoma patients who has significantly different clinical outcomes thereby may have the potential to assist clinicians in better tailoring the therapy (25). In contrast to the RATHL and the AHL2011 LYSA trials that used the ABVD regimen, the HD18 trial used a more intense regimen (escBEACOPP) and failed to show the significance of baseline metabolic parameters as a prognostic marker (22). This could be explained by a high rate of complete metabolic response achieved by using escBEACOPP. 

 One of the limitations of using MTV and TLG is the lack of standardization of the methodology, thus affecting its reproducibility. Therefore, we used both the fixed and percentage threshold approach to compute the MTV and the fixed threshold method turned out to be a more robust marker for prognosticating the disease as it independently predicted the clinical endpoint (5-year EFS) on multivariate analysis using the cox model. On the contrary, we found that TLG although a product of the tumor burden and metabolic activity could not predict 5-year EFS. It is difficult to hypothesize the reason for same, we feel that it could be due to heterogeneity in the tumor biology, and varied histology of Hodgkin that could have governed the FDG uptake. Moreover, the inherent bias associated due to small sample size could also have played a role.

 The observations of our study are based on retrospective data, the results have given insights for utilizing the MTV from baseline FDG PET CT as a surrogate marker for prognosticating advance stage Hodgkin lymphoma and can be used to model alongside the classically known clinicopathological prognostic parameters (IPS) or individually to risk stratify the patients who could develop early recurrence and thus tailor the treatment accordingly. 

 Our study is not devoid of limitations, firstly is retrospective nature. A Secondly higher number of patients were excluded which we attribute to strict selection criteria in terms of availability of blood tests at baseline (for calculating IPS) and uniform acquired PET scans. These limitations could create a selection bias and negatively affect the results. Hence prospective studies in a larger and homogeneous patient cohort treated uniformly are suggested for further validation and acceptance of these observations.

## Conclusion

The results of the present study high light the prognostic value of Metabolic Tumor Volume (WBMTV2.5) in patients with advanced-stage Hodgkin Lymphoma treated uniformly with combined modality therapy. WBMTV may have a value as a surrogate prognostic marker complementing the traditional clinical prognostic markers for advanced Hodgkin Lymphoma patients. This metabolic marker can be derived from the traditional clinical PET scans and incorporating it could risk stratify advanced Hodgkin Lymphoma patients and in foreseeable future may help clinicians in tailoring the therapeutic strategy.

## Conflict of Interest

 Authors declare no conflict of interest. 
